# A hybrid deep learning framework for automated treatment planning in cervical cancer radiotherapy

**DOI:** 10.1002/acm2.70806

**Published:** 2026-09-25

**Authors:** Haiyan Jiang, Zengtai Yuan, Zihao Liu, Yuxiang Wang, Wei Hu, Bing Yan, Yidong Yang

**Affiliations:** ^1^ Department of Engineering and Applied Physics University of Science and Technology of China Hefei Anhui China; ^2^ Ion Medical Research Institute University of Science and Technology of China Hefei Anhui China; ^3^ Department of Radiation Oncology, Division of Life Sciences and Medicine, the First Affiliated Hospital of USTC University of Science and Technology of China Hefei Anhui China

**Keywords:** automated treatment planning, cervical cancer, fluence map generation, GPU dose calculation, intensity‐modulated radiation therapy

## Abstract

**Background:**

Radiation therapy treatment planning is a time‐consuming trial‐and‐error process, and the plan quality is heavily dependent on planners' experiences, resulting in a strong demand for automated planning methods that can rapidly generate uniformly high‐quality plans.

**Purpose:**

This study aimed to develop a hybrid automated deep learning‐based plan optimization (HALO) framework, integrating deep learning fluence prediction and optimization, multileaf collimator (MLC) sequencing, and GPU‐accelerated Monte Carlo dose computation. HALO was designed to ensure robust plan generation for cervical cancer intensity‐modulated radiation therapy (IMRT).

**Methods:**

The proposed HALO framework incorporated several functional modules. First, a dose‐guided Fluence Prediction Network (DG‐FPN) was developed. A total of 120 cervical cancer clinical IMRT plans were collected to train the DG‐FPN, of which 90 plans were assigned for training, 10 for validation, and 20 for testing. The neural network took patients' computed tomography (CT) anatomy as input and predicted the 3D dose distribution and fluence maps. Next, the predicted fluence maps were further optimized to meet dose‐volume constraints (DVCs), and then the refined fluence maps were converted into deliverable segments using an MLC sequencing algorithm. Finally, dose calculation was performed using a GPU‐accelerated Monte Carlo engine. The 20 patients in the testing set were used to evaluate the HALO method, and the plan deliverability was validated by patient‐specific IMRT Quality Assurance (QA).

**Results:**

The DG‐FPN achieved superior fluence prediction accuracy compared to previous work, with a median mean absolute error (MAE) of 0.055 and a structural similarity index (SSIM) of 0.94. The automated framework generated high‐quality plans and reduced dose to adjacent OARs, with V_50Gy_ decreased from 46.5 ± 5.0% to 41.5 ± 4.8% (*p* = 0.008)for bladder, V_35Gy_ decreased from 31.5 ± 8.2% to 29.2 ± 7.5% (*p* = 0.024) for small intestine, while preserving PTV homogeneity and conformity. Importantly, the entire treatment planning time was within 3 minutes, with fluence optimization time decreased by an average of 82.6% after using deep learning prediction. The mean gamma passing rate under the 2%/2 mm criterion for the patient‐specific IMRT QA achieved 97.37 ± 1.01%.

**Conclusions:**

This study demonstrated the clinical feasibility of the proposed HALO framework for cervical cancer radiotherapy in producing high‐quality and deliverable IMRT plans. The proposed automated paradigm can serve as a stand‐alone platform for treatment planning.

## 1 INTRODUCTION

As a prevalent malignancy for women's health, cervical cancer remains a critical issue on the global public health agenda^1^. Radiation therapy is an indispensable part of the integral management of locally advanced cervical cancer, either as a primary curative treatment or as an adjuvant treatment component ^2^. The evolution of radiotherapy techniques has substantially improved treatment precision, leading to advanced modalities such as intensity‐modulated radiation therapy (IMRT)^3^, ^4^. IMRT allows for the spatial modulation of photon fluence, facilitating dose escalation to irregularly shaped targets while minimizing exposure to adjacent organs at risk (OARs)^5^, ^6^. IMRT planning requires three core components^7^: (1)Fluence map generation: calculate the x‐ray beam intensity map that can deliver the desired dose distribution to the patient; (2) MLC Sequencing: compute MLC sequences that can achieve the given fluence map; (3) Dose computation: calculate dose distribution based on the MLC sequences.

Over the past decades, plan optimization strategies have evolved from traditional analytical optimization to automated plan generation. Traditional methods, relying on gradient‐based algorithms, are clinically robust by strictly meeting dose‐volume constraints (DVCs), but suffer from long planning times^8^, ^9^. To improve efficiency, automated approaches such as Knowledge‐Based Planning (KBP) were introduced^10^, ^11^, ^12^, ^13^, ^14^. KBP models predict Dose‐Volume Histogram parameters to guide plan optimization, but the lack of spatial dose information may lead to suboptimal solutions. A faster approach is to directly predict the beam fluence maps^15^, ^16^, ^17^, ^18^. A previous study introduced the Coarse‐to‐Precise Fluence Prediction Network (C2F‐FPN), which directly generates fluence maps and thus skips the inverse plan optimization process^19^.

However, there are still occasionally some outliers with unsatisfactory quality. To improve robustness, many existing pipelines adopt a two‐stage “predict‐then‐refine” strategy, and the refinement step is often implemented in various forms, such as AI‐centric learned corrections ^20^ or heuristic post‐processing^21^. Unlike conventional KBP followed by re‐optimization, which compresses the 3D dose distribution into a few aggregated DVH parameters and discards all spatial information, our DVC‐guided refinement operates on the spatially resolved fluence map and explicitly enforces DVCs directly on the fluence map, enabling precise restriction of hotspot locations.

Despite advances in direct fluence prediction^15^, ^16^, ^17^, ^18^ and refinement, current workflows remain functionally fragmented. The predicted fluence maps still need to be imported into an external Treatment Planning System (TPS) to obtain a deliverable plan^19^, ^22^, ^23^ which holds the critical downstream modules for MLC sequencing and dose calculation. This external dependence precludes a closed‐loop plan optimization and verification. Therefore, an automated planning framework that integrates the entire workflow from fluence generation to plan verification is imperative.

In this work, we introduce a Hybrid automated Learning‐based Optimization framework (HALO) to provide an efficient end‐to‐end IMRT planning solution. Building upon C2F‐FPN^19^, an intermediate deep learning (DL) step is introduced for dose prediction^15^, ^24^, and DVC‐based optimization is further incorporated to refine the predicted fluence maps. Unlike generic predict‐then‐refine approaches that rely mainly on AI‐based correction, heuristic post‐processing, or TPS‐dependent refinement, HALO performs DVC‐guided refinement directly on the spatially resolved fluence map. This approach addresses the trade‐off between planning speed and dosimetric accuracy. Particularly, a complete treatment planning framework is proposed that operates independently of any commercial TPS, integrating all components from fluence prediction to dose calculation. In addition, the clinical deliverability of HALO produced plans is validated using patient‐specific quality assurance (QA).

## 2 MATERIALS AND METHODS

### 2.1 Data

This study included 120 anonymized cervical cancer patients who were treated with seven‐field (beam angles at 30, 90, 140, 180, 220, 275, and 330 degrees) IMRT between 2018 and 2021. Each case consisted of Computed Tomography (CT) slices and RT structure contours. The contoured structures included the planning target volume (PTV) and six OARs: bladder, left and right femoral heads, small intestine, rectum, and spinal cord. For all cases, the prescription dose to the PTV was 50 Gy in 25 fractions, delivered using 6 MV photons. The dataset was randomly partitioned into training, validation, and testing sets. A detailed summary of patient and volumetric characteristics is presented in Table 1. Statistical analyses were performed using one‐way analysis of variance (ANOVA)^25^ to compare these characteristics among the three groups. No statistically significant differences were observed (*p* > 0.05) among the training, validation, and testing sets, indicating a balanced distribution of data. The testing set was kept separate and used solely for independent evaluation of the automated planning quality.

**TABLE 1:** Summary of patient characteristics in the collected dataset.

Characteristic	Overall (*n* = 120)	Training Set (*n* = 90)	Validation Set (*n* = 10)	Testing Set (*n* = 20)	*p*‐value
Age (years)	28 ‐ 84	56.5 ± 13.6	52.3 ± 17.0	57.0 ± 13.9	0.65
PTV (cm^3^)	989.5 ‐ 2001.4	1478.9 ± 248.1	1533.4 ± 240.5	1465.3 ± 241.9	0.82
Bladder (cm^3^)	45.6 ‐ 801.3	359.9 ± 204.0	401.8 ± 231.7	348.6 ± 209.8	0.87
Left femoral head (cm^3^)	10.1 ‐295.5	83.1 ± 73.1	69.9 ± 65.5	84.7 ± 72.8	0.89
Right femoral head (cm^3^)	9.5 ‐ 251.9	75.8 ± 62.9	65.1 ± 58.0	70.1 ± 60.1	0.84
Small intestine (cm^3^)	255.7 ‐5589.0	2431.1 ± 1479.8	2788.4 ± 1691.5	2410.9 ± 1490.6	0.79
Rectum (cm^3^)	20.5 ‐165.4	74.1 ± 36.1	83.9 ± 40.2	74.9 ± 37.0	0.73
Spinal cord (cm^3^)	41.1 ‐150.3	41.8 ± 39.2	46.5 ± 36.9	41.2 ± 38.0	0.93

The ground truth clinical plans for all 120 patients were manually optimized by senior medical physicists with over five years of experience using the Varian Eclipse TPS (v15.6). The dosimetric statistics of the 120 clinical plans are summarized in Table 2. To ensure high plan quality, all planning procedures followed the recommendations of the ICRU Report 83 for IMRT dose reporting and evaluation. The plans were normalized to satisfy the institutional target‐coverage criterion, requiring that the PTV V_100%_ ≥ 95% of the prescribed dose. Dose constraints for OARs were defined according to the recommendations of the Quantitative Analyses of Normal Tissue Effects in the Clinic (QUANTEC)^26^. Finally, all ground‐truth plans were reviewed and approved by radiation oncologists and had been used for actual clinical treatment.

**TABLE 2:** Dosimetric characteristics of the clinical plans (*n* = 120).

Structure	Metrics	Clinical (*n* = 120)
PTV	$D_{2\%}$(Gy)	54.3 ± 1.8
PTV	$D_{98\%}$(Gy)	48.3 ± 1.0
PTV	CI	0.955 ± 0.015
PTV	HI	0.108 ± 0.020
Bladder	V_50Gy_(%)	49.0 ± 7.0
Rectum	V_50Gy_(%)	47.0 ± 6.0
Left femoral head	V_50Gy_(%)	1.0 ± 1.5
Right femoral head	V_50Gy_(%)	1.1 ± 1.6
Small intestine	V_35Gy_(%)	34.5 ± 11.0
Spinal cord	$D_{max}$(Gy)	43.2 ± 2.5

### 2.2 Overall workflow

**FIGURE 1:**
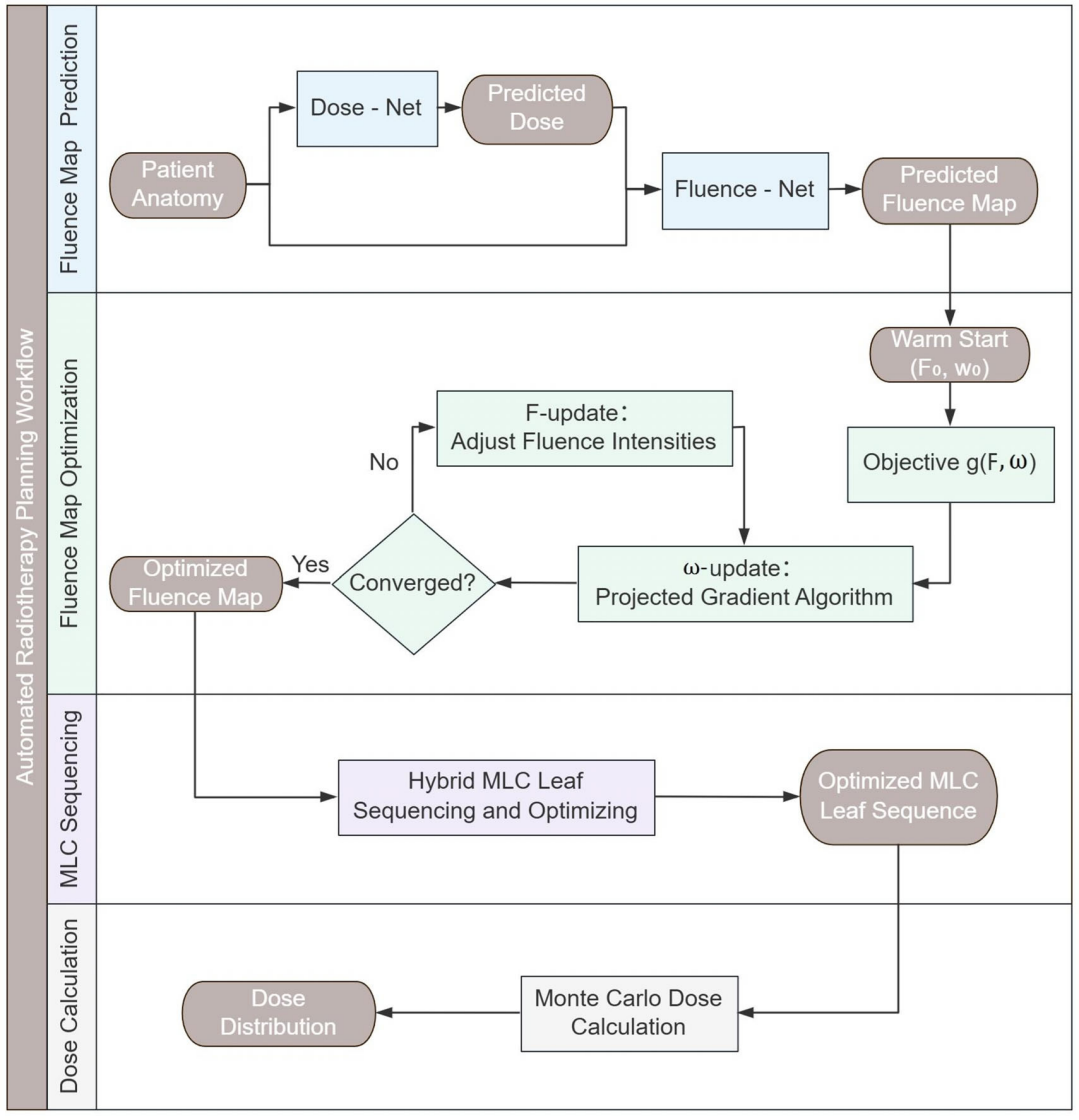
Schematic of the automated end‐to‐end IMRT planning framework.

An overview of the proposed automated treatment planning method (HALO) is shown in Figure 1. The pipeline comprises four major steps: (1) deep learning fluence prediction; (2) fluence map optimization; (3) MLC leaf sequencing; and (4) Monte Carlo dose calculation. Collectively, these steps constitute an end‐to‐end system that generates clinically deliverable IMRT plans, addressing the issue of excessive efforts in manual intervention and inconsistent plan quality determined by individual planners. The detailed pseudocode for the entire process can be found in the supplementary materials, Section S1.

### 2.3 Fluence map prediction

**FIGURE 2:**
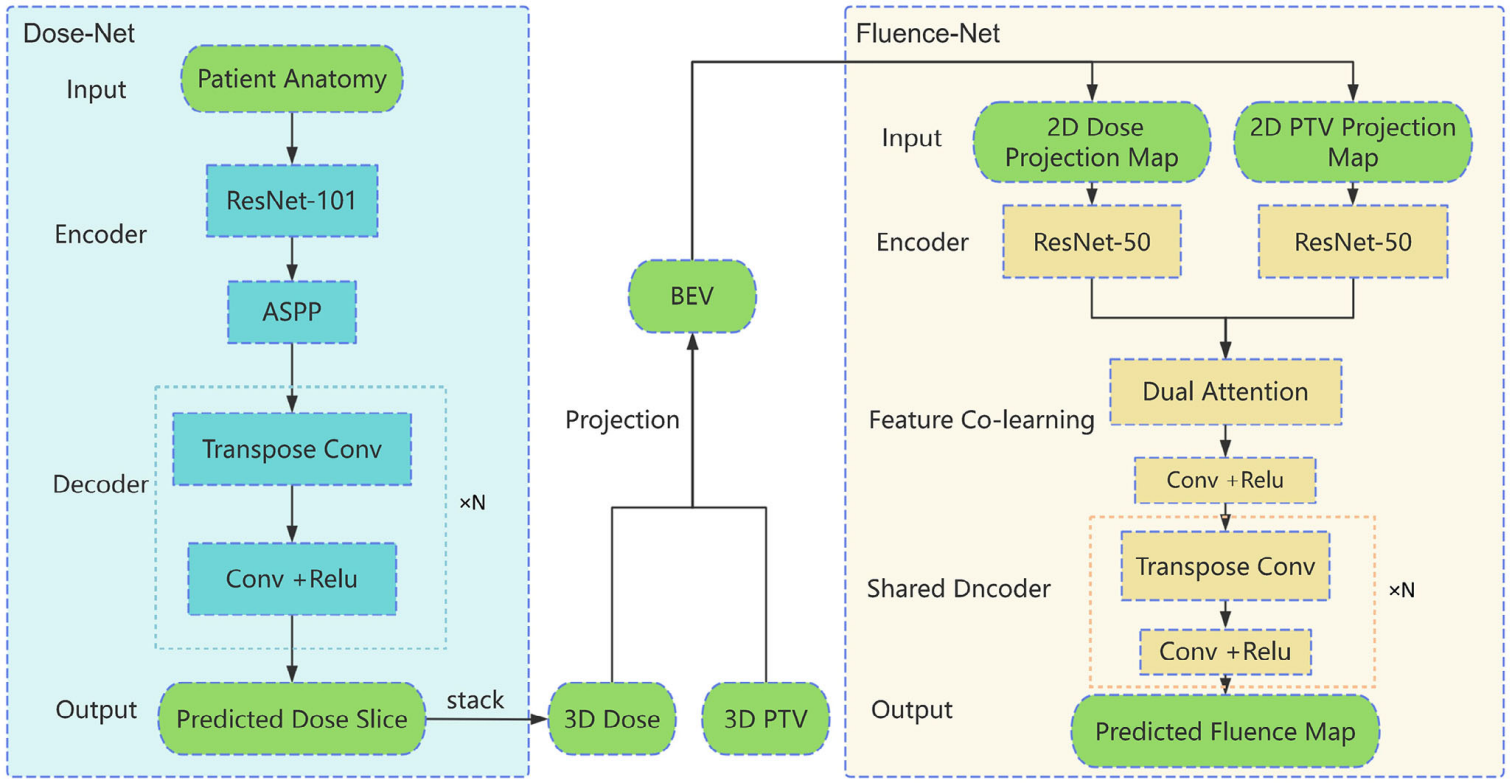
The deep learning‐based DG‐FPN framework. The pipeline takes patient anatomy as input and generates predicted fluence maps. ASPP denotes Atrous Spatial Pyramid Pooling.

In this study, we propose a Dose‐Guided Fluence Prediction Network (DG‐FPN) for automated fluence prediction (Figure 2).

In the first stage, Dose‐Net is built on a modified ResNet‐101 backbone augmented with an Atrous Spatial Pyramid Pooling (ASPP) module. The ASPP module utilizes parallel atrous convolutions with different sampling rates to capture multi‐scale contextual information. The input to Dose‐Net consists of 3D multichannel binary masks with a resolution of 256 × 256 × 7. The output is a 256 × 256 × 1 predicted dose slice. In the second stage, Fluence‐Net takes two‐channel 512 × 512 projection maps as input. The two channels correspond to the dose beam's eye view (BEV) projection and the PTV BEV projection, and the network generates a 512 × 512 predicted fluence map. The inputs are processed by dual ResNet‐50 encoders and a shared decoder to reconstruct a 2D fluence map. Furthermore, a co‐learning feature fusion module is designed to learn the conversion between dose distribution and fluence map. This dose‐guided supervision uses the predicted dose as an intermediate target, thereby improving the accuracy and fidelity of fluence map reconstruction. Dose‐Net was trained for 400 epochs using MSE loss with the Adam optimizer (batch size 4, initial learning rate 1e^−4^). Fluence‐Net was first pretrained for 200 epochs (lr 1e^−4^) and then fine‐tuned per beam field for 400 epochs (lr 6e^−5^), with a combined MAE + SSIM loss and batch size 4. Data augmentation included random translation (within ± 10 pixels) and scaling (0.8–1.2) in the transverse plane.

To evaluate the specific advantages of integrating intermediate dosimetric priors, the proposed DG‐FPN was benchmarked against C2F‐FPN^19^, a representative cascade network architecture designed for coarse‐to‐fine fluence map prediction. The prediction accuracy and structural fidelity of the synthesized fluence maps for both networks were quantitatively assessed within the same testing cohort (*n* = 20) using three complementary metrics: Mean Absolute Error (MAE) and Structural Similarity Index (SSIM) to evaluate intensity agreement and structural consistency, and the Dice Similarity Coefficient (DSC) to assess spatial overlap of the effective irradiation regions. Detailed network architectures and implementation details are provided in Section S2 of the Supplementary Materials. The architectures of Dose‐Net and Fluence‐Net are shown in Figures S1 and S2, respectively.

### 2.4 Fluence map optimization

To refine the fluence prediction results, a DVC‐based optimization module^27^ is introduced, which utilizes the DG‐FPN output as a high‐fidelity warm start. This DL‐driven initialization incorporates anatomical and dosimetric priors into the fluence representation, enabling faster and more robust convergence.

The FMO formulation^27^ is adopted, where the fluence map *F* and dose deviation variable ω constitute the primary decision variables. Owing to the non‐convexity problem, the convergence is highly sensitive to the initial state vector ($F_{0}$, $\omega_{0}$). To explore the FMO case, ($F_{0}$, $\omega_{0}$) is redefined as detailed below.

For the j‐th beam, the high‐resolution predicted fluence map (0.25cm pixel size) is denoted as $F_{j}\;\varepsilon \;R^{512\times 512}$. A down sampling function r: $\;R^{512\times 512}$→$R^{101\times 101}$ reduces the fluence map to a 0.5cm beamlet grid using block averaging or interpolation. The valid beam mask at the down sampled resolution is denoted as $M_{j}\;\varepsilon {\left\{0,1\right\}}^{101\times 101}$. $F_{0}$ is then constructed as:

(1) $$F_{0}=\sum_{j=1}^{7}\mathrm{Vec}\left(M_{j}⊙r\left(F_{j}\right)\right)$$

where ⊙ is elementwise multiplication to yield the masked fluence. V ec(·) denotes the function of flattening the matrix into a column vector. The resulting $F_{0}$ is then used to initialize $\omega_{0}$:

(2) $$\omega_{0}=proj_{\Omega }\left(AF_{0}-d^{dv}\right)$$

where, A ϵ $R^{101\times 101}$ is the dose‐influence matrix mapping the beamlet intensity vector to voxel doses. $d^{dv}$ϵ$\;R^{101}$ is the dose–volume (DV) constraint threshold vector for the structure. $\mathrm{pro}j_{\Omega }$ denotes the projection of a vector over the feasible set Ω that enforces the sparsity constraint of the DVC.

The superior initial state vector ($F_{0}$, $\omega_{0}$) directly guides the subsequent optimization, which proceeds via the following alternating updates of *F* and ω. The fluence map *F* is updated by minimizing the dose prediction error as shown in Equation (3). A step of projected gradient descent is conducted for ω as shown in Equation (4)

(3) $$F_{i+1}=\arg\min_{F\geq 0}\frac{1}{2}\left\|AF-d\left(\omega_{i}\right)\right\|\begin{array}{c} 2\\ 2 \end{array}$$

(4) $$\omega_{i+1}=proj_{\Omega_{i}}\left(\omega_{i}-\frac{\alpha }{Ln_{r}}\left(A_{r}F_{i+1}-d_{r}\right)\right)$$

where α is the step size. $Ln_{r}$ is a normalization factor, which helps ensure the stability of the optimization. The process iterates until all convergence criteria are satisfied, finally yielding the optimized fluence map $F^{*}$. This optimization strategy produces a dosimetrically superior fluence map and mitigates the risk of converging to poor local minima.

The dose–volume constraints and optimization objectives used in the FMO module are summarized in Table 3. These objectives are identical to those used in the clinical plan optimization (Section 2.1), ensuring a consistent basis for comparison between HALO‐generated plans and clinical plans. To isolate and quantitatively evaluate the dosimetric impact of the proposed deep learning (DL)‐guided initialization strategy, an ablation study was designed within the testing cohort (*n* = 20). Under identical dose evaluation settings, four distinct categories of optimization‐stage plans were compared: DG‐FPN predicted plans, where dose distributions were computed directly from the network‐predicted fluence maps without any inverse optimization; zero‐initialized optimized plans, which were optimized using standard inverse planning initialized from a zero‐state vector; DL‐guided optimized plans (the proposed method), which were optimized using the warm‐start strategy initialized from the DG‐FPN predicted fluence maps; and clinical plans, the original manually approved plans serving as the reference baseline. To evaluate computational efficiency, both the total number of optimization iterations and the absolute optimization time (in seconds) were recorded and compared between the zero‐initialized and DL‐guided optimization workflows.

**TABLE 3:** Optimization objectives and DVCs used in the FMO module and clinical plans.

ROI name	Objective type	Objective dose (Gy)	Priority
PTV	Maximum D_0%_	50	100
PTV	Minimum D_100%_	50	100
Bladder	V_45Gy_(%) < 50%	45	50
Rectum	V_45Gy_(%) < 50%	45	50
Left femoral head	V_50Gy_(%) < 5%	50	50
Right femoral head	V_50Gy_(%) < 5%	50	50
Small intestine	V_20Gy_(%) < 40%	20	50
Spinal cord	D_max _< 45Gy	45	50

### 2.5 MLC sequencing

A key component of the automated planning system is the generation of deliverable MLC apertures. First, the Engel's decomposition^28^ algorithm is applied to discretize $F^{*\;}$ into binary rectangular apertures, minimizing monitor units (MU) and the number of segments (NS) while preserving fluence fidelity. The proposed MLC decomposition can be expressed as:

(5) $$F=\sum_{K=1}^{K}\mu_{k}S^{k}\mathrm{with}\;\mu_{k}\in N_{0},S^{k}\in S,\forall k\in \left[K\right]$$

where kε{1,…K} indexes the segments $.\;\mu_{k}$ are the coefficients corresponding to each aperture and $S^{k}$ are the binary aperture configurations for each segment. The objective functions for MU and NS are:

(6) $$\mathrm{MU}=\min\sum_{k=1}^{K}\mu_{k}$$

(7) NU=minK

To further improve delivery efficiency, the LKH‐based optimization^29^, ^30^is applied to minimize the third objective, the Leaf Travel Index (LTI), thereby refining leaf‐motion trajectories. The LTI is defined as:

(8) $$\mathrm{LTI}=\min\sum_{k=1}^{K}\mu \left(S^{\pi \left(k\right)},S^{\pi \left(k+1\right)}\right)$$

(9) $$\mu \left(S^{\pi \left(k\right)},S^{\pi \left(k+1\right)}\right)=\max_{1\leq q\leq m}\left\{\left|l_{i}^{k+1}-l_{i}^{k}\right|,\left|r_{i}^{k+1}-r_{i}^{k}\right|\right\}$$

where *m* is the number of leaf pairs, and $l_{q}^{k}$, $r_{q}^{k}$ are the left and right leaf positions of pair *q* in segment *k*. and μ equals the largest single‐leaf movement required to switch from $S^{k}$ to $S^{k+1}$. In this work, we combine the hybrid sequencing method to obtain a solution for deliverable MLC apertures with higher accuracy and shorter machine movement time.

To evaluate the performance of the proposed hybrid MLC sequencing strategy, three representative algorithms, namely the Xia^31^, Siochi^32^, and Engel^28^ methods, were implemented as reference baselines. These methods represent the technical evolution from early intensity decomposition to constraint‐aware and multi‐objective optimization. While the Xia and Engel algorithms primarily focus on minimizing total MU and NS via matrix decomposition, the Siochi algorithm functions as a delivery‐driven approach optimized to control leaf motion trajectories and mechanical constraints. By incorporating the LKH‐based trajectory optimization onto Engel's heuristic foundation, the proposed hybrid MLC method aims to achieve a globally optimized trade‐off that concurrently minimizes MU, NS, and LTI. Complexity metrics and delivery efficiency across all seven beam angles were quantitatively compared among these four methods using paired t‐tests, with a two‐sided *p*‐value < 0.05 considered statistically significant.

### 2.6 Monte Carlo dose calculation

Finally, the deliverable MLC leaf sequences from the preceding stage were used to calculate the final 3D dose distribution. This was accomplished using a GPU‐accelerated Monte Carlo dose engine. The dosimetric accuracy of this Monte Carlo engine has been extensively benchmarked and validated previously^33^. The simulation modeled a 6 MV photon spectrum from a clinical linac, including detailed source characteristics and MLC transmission. A Varian Millennium 120‐leaf MLC was modeled, comprising 40 central leaf pairs with 5 mm width and 20 peripheral leaf pairs with 10 mm width.

The final deliverable HALO plans after MLC sequencing were calculated using the GPU‐accelerated Monte Carlo dose engine. To comprehensively evaluate the plan quality, the MC‐calculated dose distributions were compared with the original clinical plans within the same independent testing cohort (*n* = 20) using paired t‐tests. Dosimetric endpoints included target parameters for the PTV D_2%_, D_98%_, conformity index (CI), and homogeneity index (HI), alongside specific OAR sparing metrics including bladder V_50Gy_, rectum V_50Gy_, left and right femoral head V_50Gy_, small intestine V_35Gy_, and spinal cord Dmax. An explanation of the clinical metrics is provided in the Supplementary Materials, Section S3.

### 2.7 Patient‐specific QA

To evaluate the deliverability of the HALO‐generated plans, patient‐specific IMRT QA was performed for all 20 testing cases. The HALO‐generated MLC leaf sequences and monitor units were imported into the Varian Eclipse TPS (v15.6) and delivered on a clinical linear accelerator equipped with a Varian Millennium 120‐leaf MLC. The measured and calculated dose distributions were compared using global gamma analysis with a 2%/2 mm criterion and a 10% dose threshold, where a gamma passing rate greater than 95% was considered clinically acceptable. Statistical comparisons of the gamma passing rates were performed between the HALO plans and their corresponding clinical baseline plans.

## 3 RESULTS

### 3.1 Fluence map prediction

**FIGURE 3:**
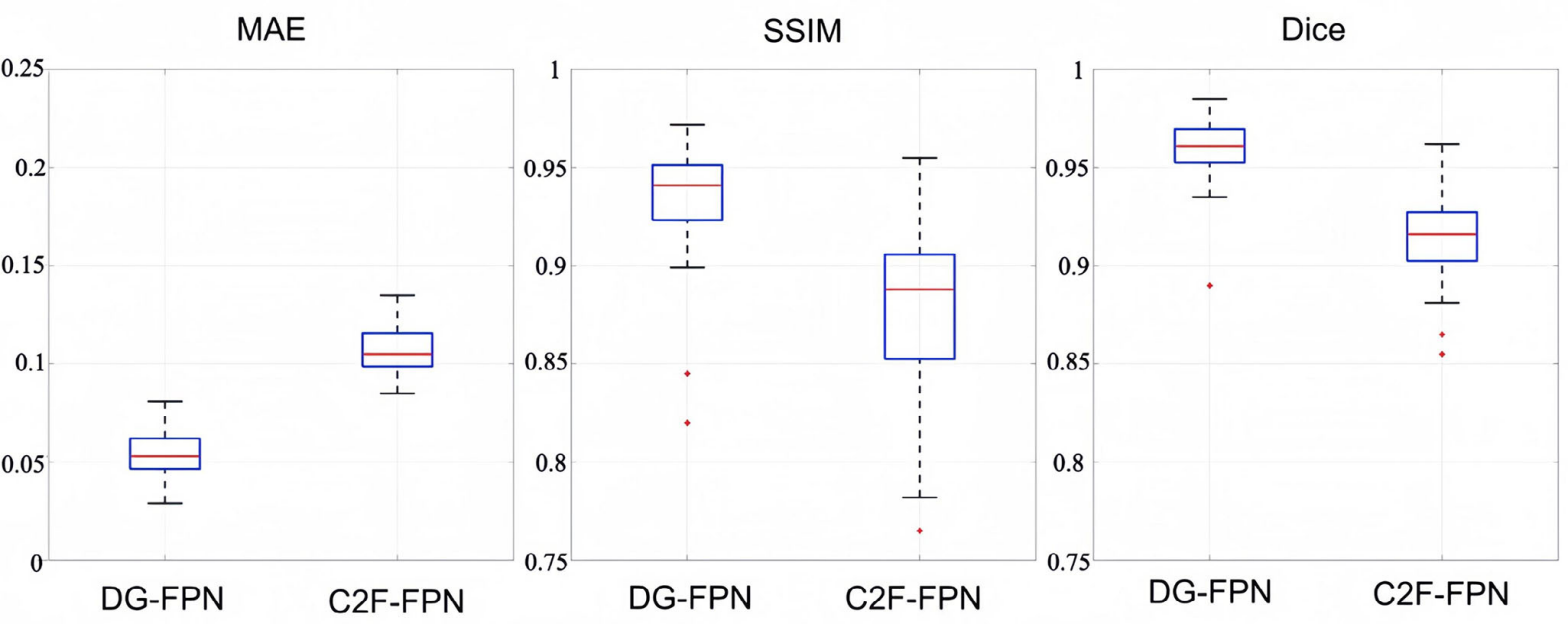
Illustration of the DG‐FPN and C2F‐FPN under different metrics. Lower MAE values, higher SSIM and Dice values indicate better performance.

A quantitative comparison of the fluence map prediction performance between DG‐FPN and C2F‐FPN^19^ is presented in Figure 3. The proposed DG‐FPN achieved a median Mean Absolute Error (MAE) of 0.055, a median Structural Similarity Index (SSIM) of 0.94 and a Dice similarity coefficient of 0.97, outperforming C2F‐FPN. In terms of different metrics, the narrower interquartile ranges for the DG‐FPN indicate better stability over the testing set.

**FIGURE 4:**
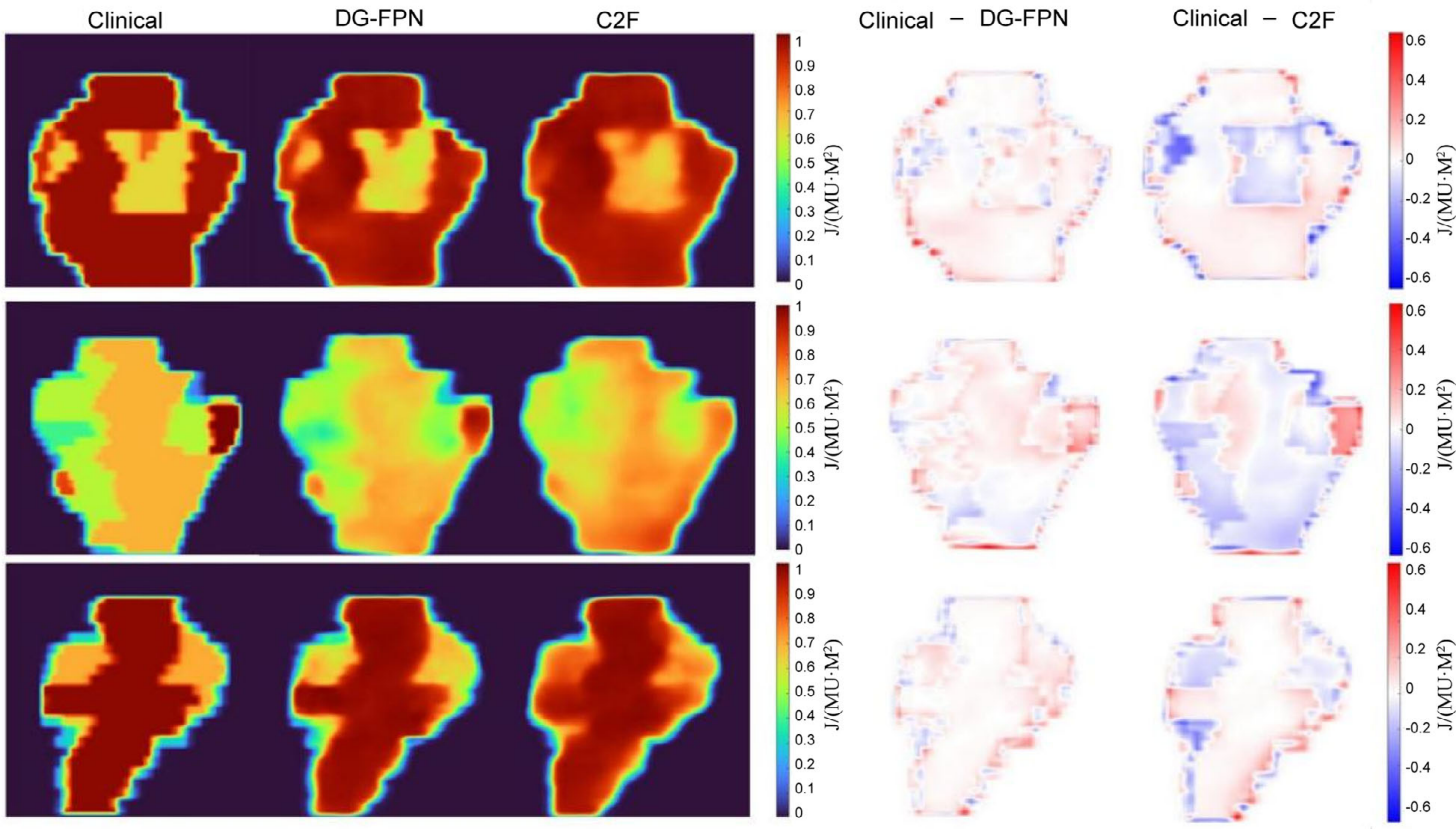
Qualitative comparison of clinical fluence maps and predictions from DG‐FPN and C2F‐FPN at beam angles 30°, 140° and 275°. The x‐axis (left to right) shows: clinical map, DG‐FPN prediction, C2F‐FPN prediction, Clinical−DG‐FPN difference, and Clinical−C2F‐FPN difference. The y‐axis lists three different beam angles.

Figure 4 provides a qualitative comparison between the clinical fluence maps and the predictions from DG‐FPN and C2F‐FPN. All fluence maps were normalized in MU and visualized using a consistent colormap. Overall, the predictions from two deep learning‐based models closely approximated the clinical fluence maps. However, as shown in the difference (fourth and fifth columns), distinct error characteristics were revealed: C2F‐FPN showed widespread deviations, whereas the DG‐FPN model's errors were primarily localized to high‐gradient regions. Despite these localized errors, the prediction from the proposed DG‐FPN model closely matched intensity patterns, and the remaining systematic deviations were within an acceptable range.

### 3.2 Fluence map optimization

**FIGURE 5:**
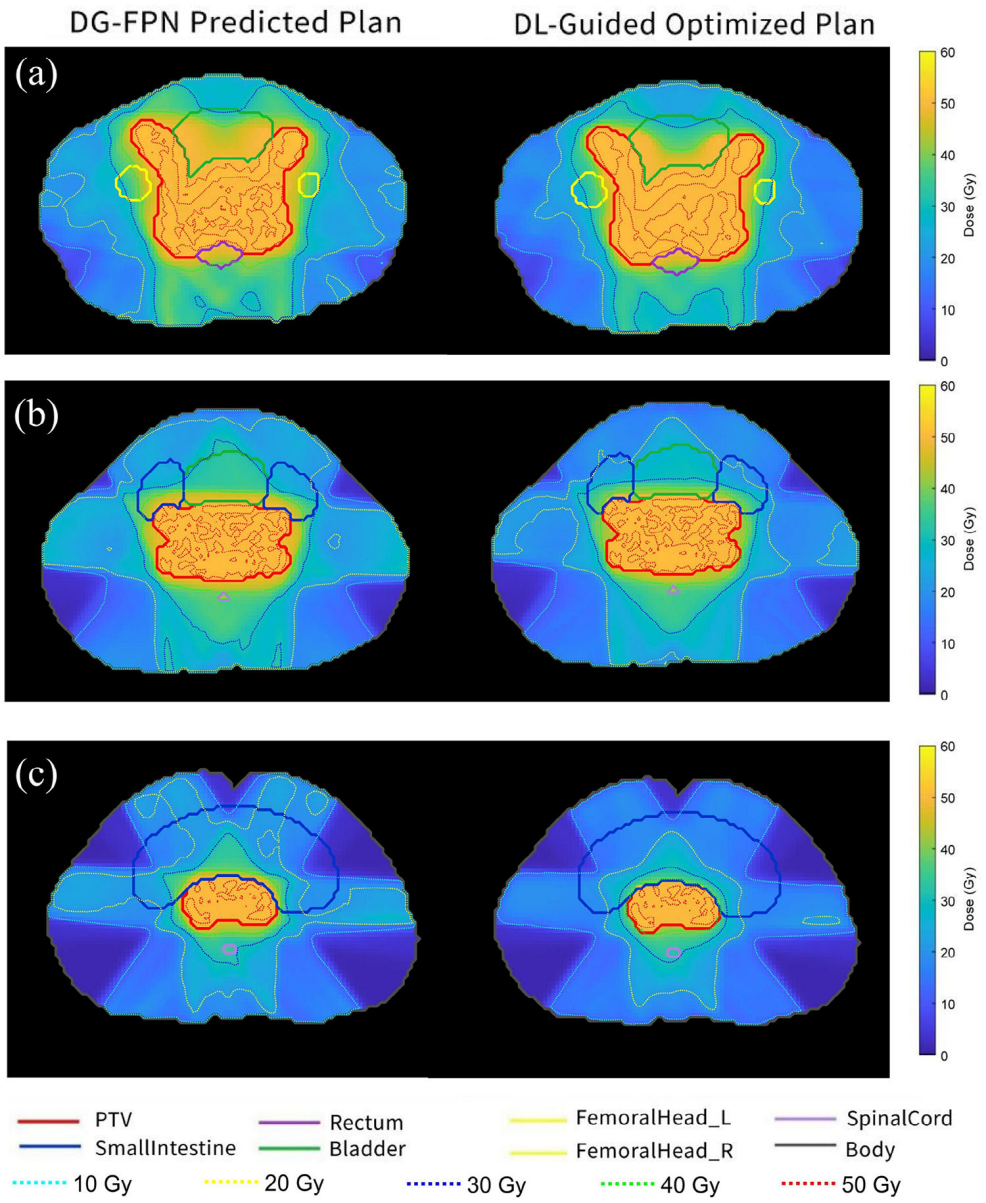
Dosimetric impact of the optimization step shown for three representative axial slices from a case. The top row highlights a substantially reduced dose to the bladder. The second row shows better dose conformity, with the prescription dose fitting the PTV more closely. The bottom row demonstrates a reduction of high‐dose spillage into the small intestine.

Figure 5 compares the dose distribution in the DG‐FPN Predicted Plan (dose computed directly from DG‐FPN without optimization) and the DL‐Guided Optimized Plan (dose optimized from the DG‐FPN predicted fluence). The optimized results improved the PTV dose conformity and reduced dose to adjacent OARs.

**FIGURE 6:**
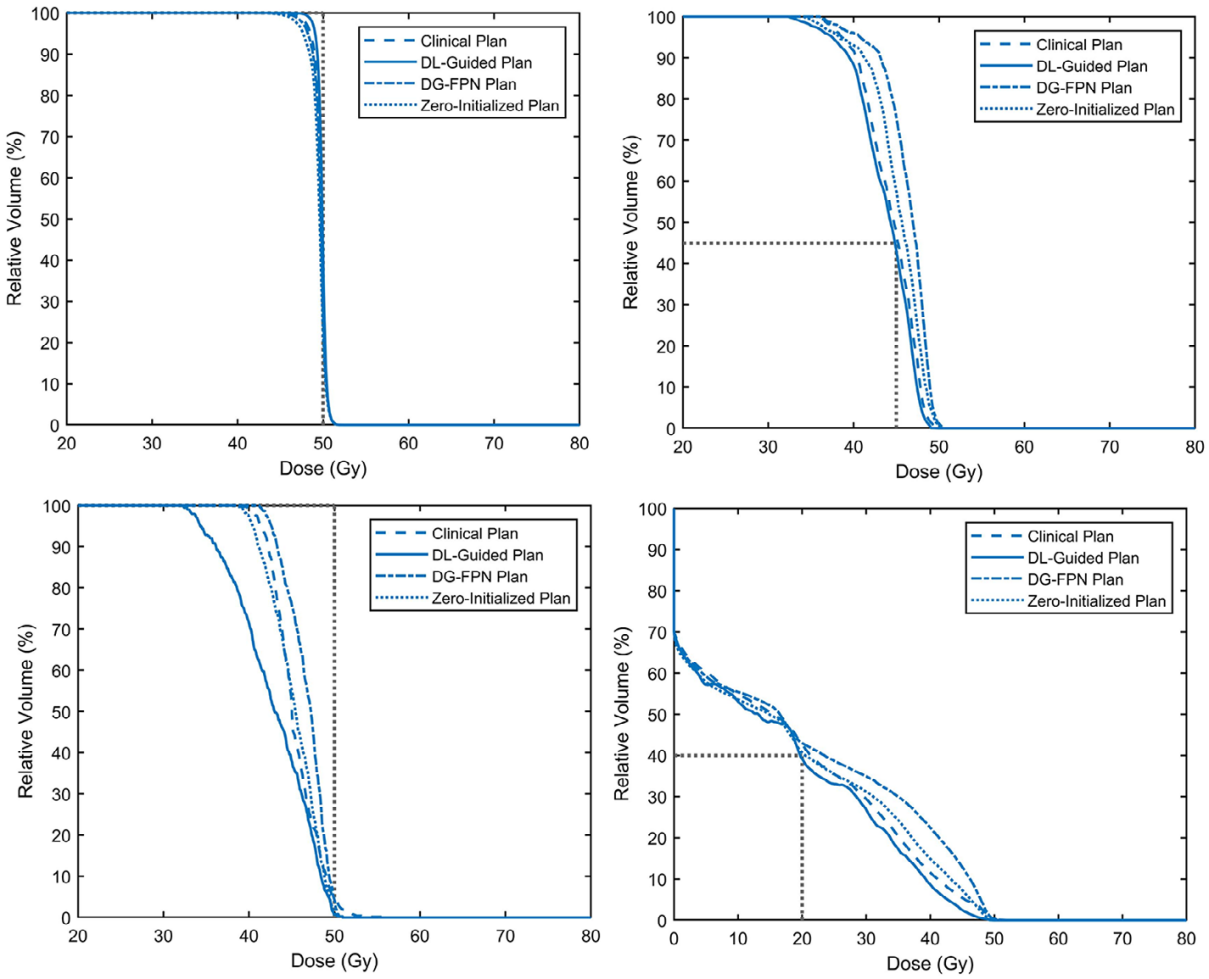
DVHs for a representative case comparing the clinical plan, DG‐FPN predicted plan, zero‐initialized optimized plan, and DL‐guided optimized plan. Subplots show DVHs for the (a) PTV, (b) bladder, (c) rectum, and (d) small intestine. The dotted lines represent the upper dose–volume constraint boundaries for each OAR (e.g., for small intestine: V20Gy(%) < 40%).

Table 4 summarizes the optimization‐stage dosimetric comparison among the clinical plans, DG‐FPN predicted plans, zero‐initialized optimized plans, and DL‐guided optimized plans. The DL‐guided optimized plans maintained PTV D_98%_ and D_2%_ comparable to the clinical plans (*p *> 0.05), while showing reduced bladder V_45Gy_ (51.2% ± 7.6% vs. 56.0% ± 8.2%, *p *= 0.032)and small intestine V_20Gy_ (35.6% ± 10.4% vs. 39.7% ± 10.9%, *p *= 0.047) compared with the clinical plans. Compared with the DG‐FPN predicted and zero‐initialized optimized plans, the DL‐guided optimized plans also showed lower OAR dose metrics, indicating the benefit of DL‐guided initialization. Figure 6 illustrates a representative case. For the PTV (Figure 6a), all plans achieved clinically acceptable target coverage. For OARs (Figure 6b–d), the DL‐Guided Optimized Plan showed the best performance. For example, the small intestine V_20Gy_ was 38% in the DL‐Guided Optimized Plan, compared with 40% in the Zero‐Initialized Optimized Plan and 43% in the DG‐FPN Predicted Plan.

**TABLE 4:** Optimization‐stage dosimetric comparison of clinical plans, DG‐FPN predicted plans, zero‐initialized optimized plans, and DL‐guided optimized plans in the testing cohort (*n* = 20).

Structure	Metrics	Clinical Plan	DG‐FPN Predicted plans	Zero‐Initialized optimized plans	DL‐guided optimized plans	*p*‐value (DG‐FPN vs Clinical)	*p*‐value (Zero‐Initialized vs Clinical)	*p*‐value (DL‐Guided vs Clinical)
PTV	D98% (Gy)	48.4 ± 0.6	48.0 ± 0.6	48.3 ± 0.5	48.4 ± 0.5	0.09	0.42	0.86
PTV	D2% (Gy)	50.6 ± 0.5	50.6 ± 0.4	50.7 ± 0.5	50.6 ± 0.4	0.91	0.58	0.88
Bladder	V45Gy (%)	56.0 ± 8.2	62.4 ± 8.7	57.1 ± 8.1	51.2 ± 7.6	0.021	0.062	0.032
Rectum	V45Gy (%)	45.2 ± 8.6	66.8 ± 9.5	55.4 ± 8.9	43.8 ± 8.3	<0.001	0.006	0.54
Small intestine	V20Gy (%)	39.7 ± 10.9	42.6 ± 11.8	40.8 ± 11.1	35.6 ± 10.4	0.23	0.65	0.047

Computational efficiency was further evaluated by comparing the proposed DL‐Guided optimization against the Zero‐Initialized optimization. The DL‐Guided optimization converged in 27 ± 8 iterations, corresponding to an optimization time of 14.7 ± 4.3 s. In contrast, the Zero‐Initialized optimization required 213 ± 27 iterations, corresponding to a time of 84.8 ± 10.9 s. The results demonstrated that the proposed warm‐start strategy improves core optimization efficiency, reducing the mean optimization time by 82.6%.

### 3.3 MLC sequencing

Plan complexity metrics for the different leaf‐sequencing algorithms are summarized in Table 5. Among the baseline algorithms, the Engel method resulted in the lowest total MU and NS, but at the cost of a high mean LTI (140.9). In contrast, the Siochi algorithm produced a low LTI (127.0). By incorporating the LKH algorithm^34^, the hybrid method successfully reduced the mean LTI to 122.9, while achieving the lowest MU and NS values. The results revealed that the hybrid MLC algorithm achieves an optimal solution.

**TABLE 5:** Plan complexity metrics for representative leaf sequencing algorithms. Lower values indicate better performance for all metrics.

Beam angle	Xia^31^			Siochi^32^			Engel^28^			Hybrid MLC		
	MU↓	NS↓	LTI↓	MU↓	NS↓	LTI↓	MU↓	NS↓	LTI↓	MU↓	NS↓	LTI↓
30	16	24	199.0	20	20	127.5	13	20	157.5	13	20	121.0
90	13	19	126.5	19	22	97.5	7	16	85.0	7	16	89.1
140	16	29	206.0	25	27	145.0	13	25	158.0	13	25	141.3
180	14	24	164.0	19	20	153.5	12	17	140.5	12	17	151.2
220	14	23	202.0	22	23	144.0	13	21	182.0	13	21	145.2
275	13	20	126.0	14	16	91.5	10	15	89.0	10	15	91.4
330	16	26	197.0	21	21	130.0	13	21	174.5	13	21	120.8
Mean	14.6	23.6	174.4	20.0	21.3	127.0	**11.6**	**19.3**	140.9	**11.6**	**19.3**	**122.9**

As detailed in Table 6, the hybrid method achieved significantly lower LTI compared to both the Siochi (*p* = 0.034) and Engel (*p* = 0.015) methods. Furthermore, the hybrid method demonstrated significant advantages in MU and NS over the Xia and Siochi algorithms (*p* < 0.05). These results statistically confirm that the hybrid MLC algorithm achieves an optimal solution by minimizing leaf travel intensity while maintaining high delivery efficiency.

**TABLE 6:** Statistical comparison (*p*‐values) of the Hybrid MLC algorithm and existing methods (Xia, Siochi and Engel).

Comparison Pair	MU	NS	LTI
Hybrid vs. Xia	0.002	< 0.001	< 0.001
Hybrid vs. Siochi	< 0.001	0.012	0.034
Hybrid vs. Engel	1.000*	1.000*	0.015

### 3.4 Dose distribution

**FIGURE 7:**
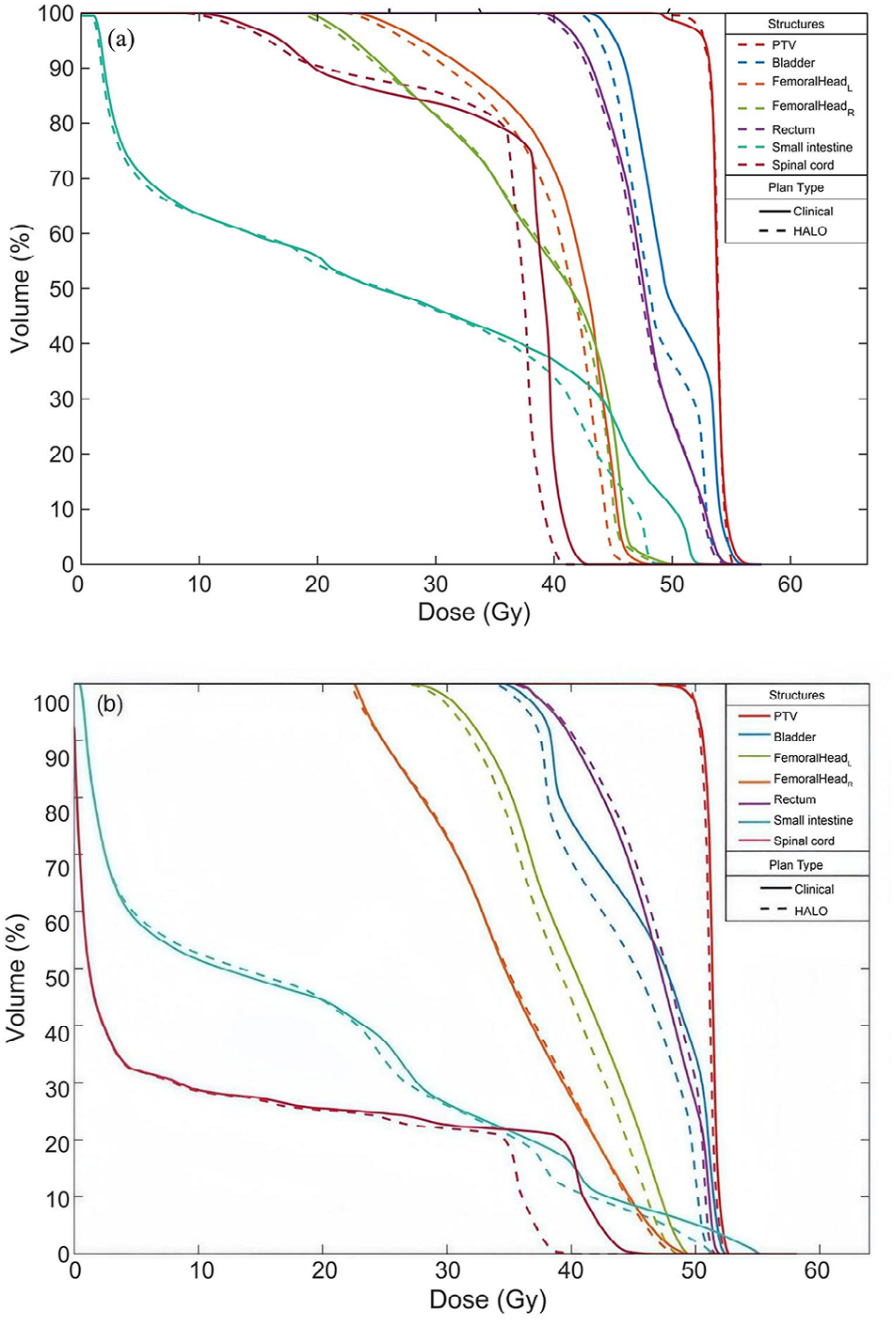
Two cases of DVHs comparison between the HALO Plan (dashed lines) and the clinical plan (solid lines).

**FIGURE 8:**
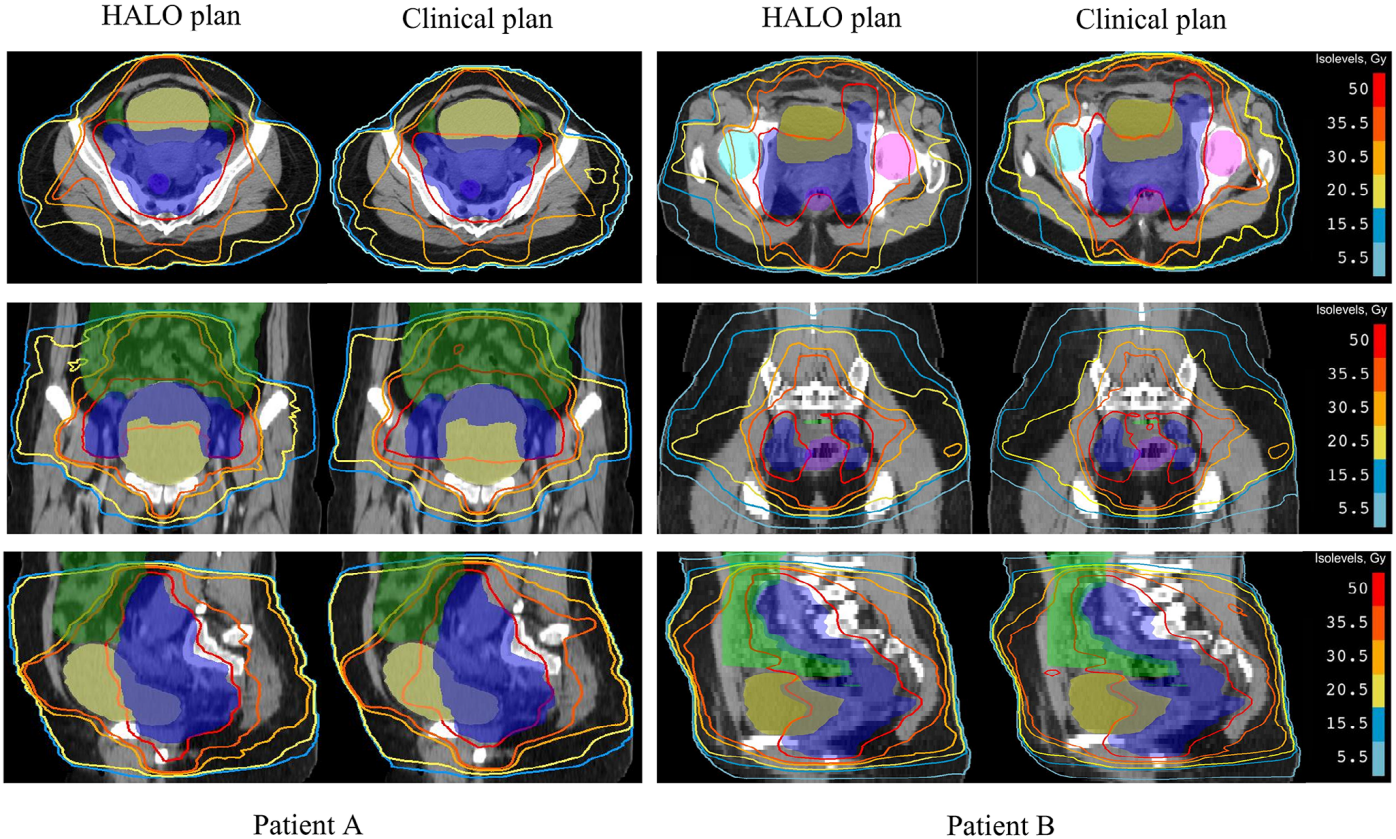
Comparison of isodose distributions between the HALO and clinical plans in the axial, coronal, and sagittal planes for two representative patients.

Figure 7 and Figure 8 present the DVHs and MC‐calculated isodose comparisons from two cases. In Figure 7, the DVH curves for the HALO and the clinical plans are comparable, with equivalent target coverage. For the bladder and rectum, HALO achieved superior results. Based on Figure 8, a visual inspection of the isodose maps revealed two notable differences consistent across both patients: (1) The clinical plan generated notable high‐dose hot spots, while the HALO plan substantially mitigated them. (2) the HALO exhibits a steeper dose fall‐off, as indicated by a smaller distance between the 50Gy and 35.5Gy isodose lines.

The dosimetric comparison between the clinical plans and HALO plans for the 20 patients is summarized in Table 7. For the PTVs, there is no statistically significant difference observed for target metrics. The primary advantage of the HALO plans was observed in OARs. Specifically, HALO plans achieved a statistically significant dose reduction (*p* < 0.05) for the bladder and the small intestine when compared with the clinical plans.

**TABLE 7:** MC calculated dosimetric comparison of plan quality metrics for the entire testing cohort.

Structure	Metrics	Clinical	HALO	*p*‐value
PTV	$D_{2\%}$ (Gy)	54.1 ± 1.5	54.0 ± 1.6	0.85
PTV	$D_{98\%\;}$(Gy)	48.5 ± 0.8	48.6 ± 0.7	0.79
PTV	CI	0.961 ± 0.008	0.962 ± 0.007	0.65
PTV	HI	0.102 ± 0.015	0.100 ± 0.016	0.58
Bladder	V_50Gy_ (%)	46.5 ± 5.0	41.5 ± 4.8	**0.008**
Rectum	V_50Gy_ (%)	45.5 ± 4.8	45.2 ± 4.5	0.72
Left femoral head	V_50Gy_ (%)	0.5 ± 0.8	0.2 ± 0.4	0.38
Right femoral head	V_50Gy_ (%)	0.6 ± 0.9	0.3 ± 0.5	0.45
Small intestine	V_35Gy_ (%)	31.5 ± 8.2	29.2 ± 7.5	**0.024**
Spinal cord	$D_{max\;}$(Gy)	42.8 ± 1.8	43.1 ± 1.9	0.35

### 3.5 Plan deliverability and computational efficiency

The deliverability of the HALO plans was validated via patient‐specific QA. Specifically, the HALO‐generated MLC leaf sequences and monitor units (MU) were imported into the Eclipse TPS, delivered on a linear accelerator equipped with a Varian 120‐leaf MLC, and then evaluated using patient‐specific QA, with the results summarized in Table 8. All clinical plans and the HALO plans surpassed the clinical acceptance threshold of 95%. The mean gamma passing rates were similar between HALO and clinical plans: 97.37% ± 1.01% vs. 97.29% ± 1.0. (*p* = 0.49)

**TABLE 8:** Global gamma passing rates (2%/2 mm, 10% threshold) comparing clinical plans and HALO plans.

Plan type	Mean ± SD (%)	Minimum pass rate (%)	*p*‐value
Clinical plans	97.29 ± 1.00	95.5	–
HALO plans	97.37 ± 1.01	95.6	0.49

Table 9 provides a detailed execution time breakdown of the HALO framework across its four key functional modules. The entire end‐to‐end automated pipeline completed plan generation within an average total execution time of 176.3 ± 9.9 s (under 3 minutes). Mechanistically, MLC leaf sequencing constituted the primary computational bottleneck, consuming 129.7 ± 7.9 s (73.6% of the total duration), followed by Monte Carlo dose calculation (24.8 ± 4.2 s), fluence optimization (14.7 ± 4.3 s), and deep learning‐based fluence prediction (7.1 ± 0.4 s). These performance benchmarks indicate that the modular components of HALO are computationally optimized for rapid, automated implementation.

**TABLE 9:** Module‐wise runtime of the HALO framework.

Module	Time (s)	Percentage of total time (%)
Fluence prediction	7.1 ± 0.4	4.0
Fluence optimization	14.7 ± 4.3	8.3
MLC sequencing	129.7 ± 7.9	73.6
Dose calculation	24.8 ± 4.2	14.1
Total HALO	176.3 ± 9.9	100

## 4 DISCUSSION

This study proposed and validated the HALO framework which delivers an end‐to‐end IMRT planning solution for cervical cancer. Previous studies have proposed to automatically generate direct fluence maps^15^, ^16^ and most of them ended at the fluence prediction stage. More recently, several significant studies have sought to bridge the gap toward clinical deliverability. For instance, Huang et al.^35^ successfully demonstrated an automated workflow for cervical cancer by converting deep learning dose predictions into dose rings to guide optimization within TPS, facilitating clinical adoption. Meanwhile, Heilemann et al. ^36^ introduced a transformative approach for prostate VMAT by using an encoder‐decoder network to directly predict MLC motion sequences, effectively streamlining the planning process by bypassing traditional sequencing. Although these studies demonstrated the feasibility and efficiency of automated planning, they remained dependent on commercial TPS environments. In contrast, HALO explored a full path from fluence prediction and optimization, MLC sequencing and dose computation, finally resulting in clinically acceptable treatment plans.

The core innovation of our framework lies in the predict‐then‐DVC‐guided refinement approach, which directly addresses the trade‐off between the speed of deep learning prediction and the thoroughness of conventional optimization. In contrast to KBP, which predicts DVH parameters and loses spatial information, dose‐ring optimization, which imposes only indirect constraints on fluence^35^, and direct MLC prediction, which bypasses fluence optimization^36^, our DVC‐guided refinement directly optimizes the fluence map under explicit dose–volume constraints. While DL‐based fluence prediction enables quick initialization, it can still experience occasional errors—particularly in regions with sharp dose gradients or at the edges of the field (Figures 3 and 4). These errors are most noticeable at the boundaries where high‐dose and low‐dose regions meet, where the system may struggle to maintain precision. A representative failure mode occurs when the PTV and OAR are in close proximity, resulting in an extremely steep dose gradient. Specifically, large areas of low dose may be under‐predicted, leading to inadequate dose delivery, while the borders of high‐dose regions may have excessive dose, creating unintended hotspots in adjacent OARs. Fixing these errors is crucial for clinical use, as uncorrected issues at the edges can reduce target coverage or result in too much dose to nearby sensitive organs, thereby compromising treatment safety. The DVC‐based optimization step, which converges rapidly in practice, was specifically designed to minimize these errors, particularly at the PTV–OAR transition zone (Figure 5). This finding suggests that DL‐based fluence prediction can serve as an effective warm‐start for optimization, while the refinement step provides the necessary control over dose distribution to ensure optimal target coverage and OAR sparing.

This synergistic approach also extends to the subsequent generation of the deliverable plan. A key component is the hybrid MLC sequencing algorithm, for which the underlying techniques are well established. The algorithm combines Engel's method to reduce MU and NS with the LKH heuristic to minimize LTI, ensuring high delivery efficiency(Table 5). The clinical feasibility of the entire workflow was confirmed through patient‐specific QA validation (Table 8). Critically, our HALO‐generated plans achieved a mean gamma passing rate of 97.37 ± 1.01%, exceeding standard clinical acceptance criteria, indicating that the resulting plans are clinically deliverable and comparable to manually planned results. When integrated with the GPU‐accelerated Monte Carlo dose calculation engine, the HALO framework unifies all steps—from prediction and refinement to sequencing and dose computation—into a single pipeline that is independent of any clinical TPS and can produce a dosimetrically robust, clinically deliverable IMRT plan in under 3 minutes.

Despite the promising results, this study has limitations. First, this study was based on a single‐institution cohort with a small and limited test cohort (*n* = 20) , which may limit the generalizability of our findings. Second, this study employed a single treatment protocol and lacked multi‐observer plan quality assessment. The ground‐truth plan quality inherently reflects clinical practice under clinically acceptable conditions in a single‐center setting. Third, our framework was specifically designed for cervical cancer IMRT. Its applicability to more complex anatomical sites, such as head and neck, or different modalities like VMAT, remains to be explored. Further validation in larger, multi‐center cohorts and other treatment settings is warranted to assess the robustness and generalizability of the proposed framework.

## 5 CONCLUSION

This study developed and validated a fully automated IMRT planning framework HALO for cervical cancer. The HALO plans maintained PTV coverage and conformity while significantly reducing dose to critical OARs. The “predict‐then‐DVC‐guided refinement” substantially accelerated optimization, enabling generation of a clinically robust plan in under 3 minutes. The plans were also clinically deliverable, with a mean gamma passing rate of 97.37% ± 1.01%. Collectively, the HALO framework is validated as an independent platform for automated treatment planning and plan quality evaluation, providing a systematic foundation for future clinical applications.

## AUTHOR CONTRIBUTIONS STATEMENT

Haiyan Jiang, Zengtai Yuan, and Zihao Liu designed the study, collected the data, and wrote the manuscript. Haiyan Jiang, Yuxiang Wang, and Bing Yan performed the experiments. Yuxiang Wang and Wei Hu analyzed the data and developed the algorithms. All authors approved the final version of the manuscript.

## CONFLICT OF INTEREST STATEMENT

The authors declare no conflicts of interest.

## ETHICS STATEMENT

This retrospective study was approved by the Ethics Committee of the First Affiliated Hospital of University of Science and Technology of China (USTC). The requirement for informed consent was waived by the committee due to the retrospective nature of the study.

## Supporting information




**Supporting Information**: acm270806‐sup‐0001‐SuppMat.pdf

**Supporting Information**: acm270806‐sup‐0002‐SuppMat.docx

**Supporting Information**: acm270806‐sup‐0003‐FigureS1.tif

**Supporting Information**: acm270806‐sup‐0004‐FigureS2.tif

## Data Availability

The datasets generated during and/or analyzed during this study are available from the corresponding author on reasonable request.
