# Supplementary material for: A hybrid deep learning framework for automated treatment planning in cervical cancer radiotherapy
Source: J Appl Clin Med Phys. 2026 Sep 25;27(10):e70806. doi: 10.1002/acm2.70806 (PMC13615310; doi:10.1002/acm2.70806)
Supplement: Supplementary file 1 [file ACM2-27-e70806-s003.pdf]

Pseudocode 1: Clarity of the Development, Execution, and Evaluation of HALO

Input: x = Patient Anatomy;

Output: Final deliverable plan $(S^{\pi(k)}, D_{\text{final}})$;

Notation: A : dose-influence matrix; d^{DV} : DVH thresholds;
Ω : DVC-feasible set; M_j : valid beam mask;
r : denotes a specific anatomical region of interest (ROI);

Offline stage

```
DG-FPN training;
for epoch t = 1,...,T do
    D^train ← DoseNet(X^train; θ^dose_{t-1});
    θ^dose_t ← θ^dose_{t-1} − η∇L_dose(D^train, y^label_dose);
for epoch t = 1,...,T do
    F^train ← FluenceNet(BEV_Proj(D^train, PTV^train); θ^flu_{t-1});
    θ^flu_t ← θ^flu_{t-1} − η∇L_flu(F^train, y^label_flu);
return (θ^dose_final, θ^flu_final);
```

$$D^{\text{train}} \leftarrow \text{DoseNet}(X^{\text{train}}; \theta^{\text{dose}}_{t-1})$$
$$\theta^{\text{dose}}_t \leftarrow \theta^{\text{dose}}_{t-1} - \eta \nabla L_{\text{dose}}(D^{\text{train}}, y^{label}_{\text{dose}})$$
$$F^{\text{train}} \leftarrow \text{FluenceNet}(\text{BEV\_Proj}(D^{\text{train}}, \text{PTV}^{\text{train}}); \theta^{\text{flu}}_{t-1})$$
$$\theta^{\text{flu}}_t \leftarrow \theta^{\text{flu}}_{t-1} - \eta \nabla L_{\text{flu}}(F^{\text{train}}, y^{label}_{\text{flu}})$$

Online stage

- *for patient* $x \in X^{\text{test}}$ *do*
  - **(A) DG-FPN inference**;
  - $D \leftarrow \text{DoseNet}(x; \theta^{\text{dose}}_{\text{final}})$;
  - $F \leftarrow \text{FluenceNet}(\text{BEV\_Proj}(D, \text{PTV}); \theta^{\text{flu}}_{\text{final}})$;
  - **(B) Fluence map optimization**;
  - *for beam* $j \in \mathcal{J}$ *do*
    - $F^{\downarrow}_j \leftarrow \text{Downsample}(F_j)$;
    - $F_{0,j} \leftarrow \text{vec}(M_j \odot F^{\downarrow}_j)$;
  - $F_0 \leftarrow \text{concat}(\{F_{0,j}\}_{j \in \mathcal{J}})$;
  - $w_0 \leftarrow \text{proj}_{\Omega}(AF_0 - d^{\text{DV}})$;
  - *for iteration* $i \in \mathcal{I}$, *OAR* $r \in \mathcal{R}$ *do*
    - $F_{i+1} \leftarrow \arg\min_{x \geq 0} \frac{1}{2} \|AF_i - d(w_i)\|^2_2$;
    - $w_{i+1} \leftarrow \text{proj}_{\Omega}\Big(w_i - \frac{\alpha}{Ln_r}(A_r F_{i+1} - d_r)\Big)$;
    - $i \leftarrow i + 1$;
  - return $F^{\star}$;
  - **(C) MLC sequencing**;
  - $\{S^k, \mu_k\}^K_{k=1} \leftarrow \text{EngelDecomp}(F^{\star})$;
  - $\mu(S^k, S^{k+1}) = \max_i \max\{|l^{k+1}_q - l^k_q|,\ |r^{k+1}_q - r^k_q|\}$;
  - $\pi \leftarrow \text{LKH}\big(\{S^k\}, \text{cost} = \mu\big)$;
  - $\text{LTI} = \sum^{K-1}_{k=1} \mu\big(S^{\pi(k)}, S^{\pi(k+1)}\big)$;
  - **(D) Monte Carlo dose computation**;
  - $D_{\text{final}} \leftarrow \text{MC}\big(\{(S^{\pi(k)}, \mu_{\pi(k)})\}^K_{k=1},\ \text{MLC model}\big)$

Evaluation (TPS verification);

DVH $\leftarrow$ $\{D_{\text{final}}$ vs. $D_{\text{TPS}}\}$;

$\Gamma\left\{TPS \leftarrow \{(\mathcal{S}^{\pi(k)}, \mu_{\pi(k)})\}^K_{k=1}\right\}$;

Gamma pass rate$\geq 95\%$;
