# Supplementary material for: A hybrid deep learning framework for automated treatment planning in cervical cancer radiotherapy
Source: J Appl Clin Med Phys. 2026 Sep 25;27(10):e70806. doi: 10.1002/acm2.70806 (PMC13615310; doi:10.1002/acm2.70806)
Supplement: Supplementary file 2 [file ACM2-27-e70806-s004.docx]

### **Supplementary Materials**

**S 1: Algorithm**

The overall computation of HALO is summarized in Algorithm 1. The workflow is divided into two phases: an offline training phase and an online application phase. During the training phase, Dose-Net parameters ($\text{θ}_{\text{final}}^{\text{dose}}$) are finally refined through Adam33 on the dose loss $\text{L}_{\text{dose}}$, and the Fluence-Net parameters ($\text{θ}_{\text{final}}^{\text{flu}}$) are fine-tuned in a sequential manner. For HALO online application to a new case, the trained DG-FPN network performs inference to yield a fluence map $\text{F}$, which is then optimized as an FMO problem. This process continues until convergence to the final optimized solution${\text{ }\text{F}}^{\text{∗}}$. There is a solution by integrating MLC sequencing and MC computation, via a reduced number of segments and an efficient ordering${\text{ }\text{S}}^{\text{ }\text{π}\text{(}\text{k}\text{)}}$, whereby the optimized fluence map is decomposed into a high-fidelity dose distribution $\text{D}_{\text{final}}$. Notably, HALO is designed with a modular architecture where each component is separable and replaceable. This allows intermediate outputs, such as optimized fluence maps or MLC sequences, to be independently exported and integrated into commercial TPS for further verification or joint optimization. Finally, the entire plan is clinically validated in the TPS via a gamma analysis requiring a pass rate of ≥ 95%.

**S2: Deep Learning Methodology and Implementation Details**

**S2.1: Dose-Net**

The Dose-Net, an encoder-decoder structure as illustrated in Figure 1, consists of a contracting path to extract features through stacked residual blocks and an expanding path to recover features through stacked transpose convolutions and convolution blocks. In the contracting path, Dose-Net was followed by the backbone of ResNet-101 and an atrous spatial pyramid pooling (ASPP) module was inserted at the end. In the expanding path, each layer consists of three operations: a 3×3 transposed convolution with stride 2 for up-sampling, a skip connection with the corresponding features from the contracting path, and a convolution block consisting of two 3×3 convolutions for recovering the features. To smoothly reduce the number of filters, two 1×1 convolutions were used at the end of the Dose-Net. All ReLU activations are followed by batch normalization.

Figure S1. The architecture of the proposed Dose-Net for dose prediction. Each box represents the extracted feature map. The number above each feature map refers to the number of channels.

**S 2.2: Fluence-Net**

As illustrated in Figure 2, the proposed Fluence-Net contained an encoder for the 2D dose projection input, another separate encoder for the 2D PTV projection input, a co-learning component and a shared decoder component. Both encoders were followed by the backbone of ResNet-50, aiming to extract features relevant to the input image modality. The co-learning component fused the modality-specific features from the two encoders and consisted of a dual attention block to fuse the highest level features and three convolution blocks, where each block contained two 3×3 convolutions to fuse other features. Each layer in the shared decoder component consisted of three operations: one 3×3 transposed convolution with stride 2 for up-sampling, one skip connection taking the corresponding features from the co-learning component, and one convolution block consisting of two 3×3 convolutions for recovering the features. The Fluence-Net ended with three 1×1 convolutions to smoothly reduce the number of filters. All ReLU activations are followed by batch normalization.

Figure S2. The architecture of the proposed Fluence-Net for fluence map prediction. Each box represents a feature map. The number above each feature map refers to the number of channels.

**S 2.3: Data Preprocessing**

For the Dose-Net, the structure contours and dose distribution were extracted from the TPS and converted to 3D binary mask matrixes with 256 × 256 pixels in the transverse plane. A volume of interest (VOI, 256 × 256 × 7, the channel of VOI were PTV binary mask, rectum binary mask, bladder binary mask, small intestine binary mask, spinal cord binary mask, and left and right femoral head binary masks respectively) was extracted from the same slice in all OARs 3D matrixes. The pixel intensity in the 3D dose matrix was divided by the prescription dose to scale the pixel intensity to around the [0,1] range. The paired structure and corresponding dose slices (256 × 256 × 1) were fed into the Dose-Net. The predicted dose slice outputed from the trained Dose-Net were stacked to form 3D dose for subsequent fluence map prediction.

For the Fluence-Net, the predicted 3D dose distribution and 3D PTV contours are projected to the beam’s eye view (BEV) and converted to a 2D dose projection map (512×512) and a 2D PTV projection map (512×512), respectively. The pixel intensity in the 2D dose projection map, 2D PTV projection map and fluence map was divided by the maximum value inside all the maps in the training set, and the scaled maps were fed into the Fluence-Net to accelerate the network convergence process.

**S 2.4: Data Augmentation**

To augment the training set, the paired structure and corresponding dose slices were randomly selected to undertake the following actions in the transverse plane: shifting in the left-right and up-down directions within 10 pixels and scaling in the range of 0.8 ~ 1.2.

**S 2.5: Model checkpoint**

The validation set (n=10) was used exclusively for model selection and early stopping. It was never used to fit model parameters, to tune any component on the test set, or to report final performance.

Dose-Net: Trained first. At the end of each epoch we computed validation dose MAE and validation SSIM. The best checkpoint was defined as the epoch with minimum validation MAE; SSIM acted as a tie-breaker (higher is better).

FluenceNet: Initialized with the selected DoseNet checkpoint. During the training phrase of FluenceNet , we monitored validation fluence MAE. The best FluenceNet checkpoint was chosen by the minimum validation fluence MAE with SSIM as a tie-breaker.

**S 2.6: Experimental Hyperparameters**

The Dose-Net weights were randomly initialized, and the number of training epochs was set to 400. The mean squared error (MSE) was chosen as the loss function, and the Adam algorithm was used as the optimizer to minimize the loss function with a batch size of 4 and an initial learning rate of 0.0001.

All the fluence maps and projection maps in the training set were used to pretrain the Fluence-Net for 200 epochs with an initial learning rate of 0.0001, and then each treatment field was trained individually based on the pretrained Fluence-Net for 400 epochs with an initial learning rate of 0.00006. The mean absolute error (MAE) and structural similarity (SSIM) were added with equal weights as the loss function, and the Adam algorithm was used as the optimizer to minimize the loss function with a batch size of 4.

**S 2.7: Experimental Setting**

Both two CNNs were trained with an NVIDIA GeForce RTX 3090 GPU with 24 GB memory and an Intel Core i9-11900F CPU processor with 64 GB RAM.

**S 2.8: Postprocessing**

Predicted 2D dose slices were stacked to reconstruct the 3D dose volume. The predicted 512×512 fluence maps were rescaled to their original intensity and then down-sampled to a 101×101 grid resolution to match the clinical resolution required by the Treatment Planning System.

**S 3: Metrics**

For plan evaluation, to assess the PTV coverage, $\text{D}_{\text{98\%}}$, $\text{D}_{\text{2\%}}$ (dose received by 98%, 2% of the PTV volume), homogeneity index $\text{HI}$, and conformity index $\text{CI}\text{ }$were calculated as follows:

$$\text{HI=}\text{(D}_{\text{2\%}}\text{−}\text{D}_{\text{98\%}}\text{)/}\text{D}_{\text{50\%}}$$

$$\text{CI=}{\text{(V}_{\text{PTV}}\text{⋂}\text{V}_{\text{100\%}}\text{)}}^{\text{2}}\text{/}\text{(V}_{\text{PTV}}\text{×}\text{V}_{\text{100\%}}\text{)}$$

where $\text{V}_{\text{PTV}}$ represents the volume of the PTV and $\text{V}_{\text{100\%}}$ is the volume encompassed by the 100% isodose line.

To assess OAR sparing, we computed V_50_, defined as the percentage of the organ’s volume receiving at least 50 Gy (unit: %, lower indicates better sparing), and Dmax, defined as the maximum dose within the organ (unit: Gy, lower indicates better sparing).

**DSC calculation for fluence maps**

Since fluence maps are continuous-valued intensity distributions rather than binary masks, the Dice similarity coefficient (DSC) was computed after thresholding. Specifically, both predicted and ground-truth fluence maps were normalized to [0,1], and pixels with intensity of 10% were defined as the active fluence region. Binary masks were then generated as

Bpred=(Pred>0.1) Bgt=(GT>0.1),

The DSC was calculated as

DSC =$\frac{\text{2|Bpred ⋂Bgt|}}{\text{|Bpred|+|Bgt|}}$,

For rare cases where both binary masks were empty, the DSC was undefined because the denominator was zero; therefore, the value was recorded as NaN (Not a Number).
